# Cyclin G2 Suppresses Estrogen-Mediated Osteogenesis through Inhibition of Wnt/β-Catenin Signaling

**DOI:** 10.1371/journal.pone.0089884

**Published:** 2014-03-03

**Authors:** Jinlan Gao, Qi Liu, Xing Liu, Chunyan Ji, Shengqiang Qu, Shusen Wang, Yang Luo

**Affiliations:** The Research Center for Medical Genomics, Key Laboratory of Cell Biology, Ministry of Public Health, Key Laboratory of Medical Cell Biology, Ministry of Education, China Medical University, Shenyang, China; Georgia Regents University, United States of America

## Abstract

Estrogen plays an important role in the maintenance of bone formation, and deficiency in the production of estrogen is directly linked to postmenopausal osteoporosis. To date, the underlying mechanisms of estrogen-mediated osteogenic differentiation are not well understood. In this study, a pluripotent mesenchymal precursor cell line C2C12 was used to induce osteogenic differentiation and subjected to detection of gene expressions or to manipulation of cyclin G2 expressions. C57BL/6 mice were used to generate bilateral ovariectomized and sham-operated mice for analysis of bone mineral density and protein expression. We identified cyclin G2, an unconventional member of cyclin, is involved in osteoblast differentiation regulated by estrogen *in vivo* and *in vitro*. In addition, the data showed that ectopic expression of cyclin G2 suppressed expression of osteoblast transcription factor *Runx2* and osteogenic differentiation marker genes, as well as ALP activity and *in vitro* extracellular matrix mineralization. Mechanistically, Wnt/β-catenin signaling pathway is essential for cyclin G2 to inhibit osteogenic differentiation. To the best of our knowledge, the current study presents the first evidence that cyclin G2 serves as a negative regulator of both osteogenesis and Wnt/β-catenin signaling. Most importantly, the basal and 17β-estradiol-induced osteogenic differentiation was restored by overexpression of cyclin G2. These results taken together suggest that cyclin G2 may function as an endogenous suppressor of estrogen-induced osteogenic differentiation through inhibition of Wnt/β-catenin signaling.

## Introduction

Postmenopausal osteoporosis (PMOP) is characterized by the reduction in bone mass after menopause in women accompanied with a dramatic shift of adipocyte/osteoblast ratio in the bone [1], [2]. PMOP may increase bone fragility and the susceptibility to bone fractures, which have become a major health concern worldwide [3]. Estrogen could be an important factor in the maintenance of bone mass, and its deficiency contributes to PMOP development [4], [5]. Normal levels of estrogen aid in maintaining the balance between osteoblastic bone formation, osteoclastic bone resorption and adipocyte formation by regulating multiple cell signals to coordinate distinct cellular functions [6], [7]. However, the underlying mechanisms by which estrogen induces bone formation are yet to be determined.

Estrogen provides several functions in the regulation of cell growth and differentiation through estrogen receptor (ER), which is expressed in various types of cells including mesenchymal stem cells [8]–[10]. During estrogen activation of cell functions, estrogen molecules bind and activate ER to regulate expression of downstream genes to perform cellular functions such as regulating osteogenesis [11]. Previous studies demonstrated the expression of cyclin G2 is rapid and robustly repressed by estrogen through an ER-dependent way [12]. Cyclin G2 is an unconventional cyclin that belongs to the cyclin G family, which also comprises cyclin G1 and cyclin I [13], [14]. Expression of cyclin G2 fluctuates during the cell cycle with a peak in the late S/early G_2_ phase, and atypically increases during cell cycle arrest and apoptosis [15]–[17]. Our previous work also showed that cyclin G2 is a negative regulator in gastric cell proliferation [18], [19]. Recently, cyclin G2 was shown to induce adipose differentiation, which is considered as the opposite to osteogenic differentiation [20], through the peroxisome proliferator-activated receptor γ (PPARγ) pathway [21]. These data suggest that cyclin G2 serves a potential role in bone formation and may be involved in the development of PMOP.

Osteogenic differentiation of mesenchymal stem cells is a complicated process characterized by expression of the main transcription factor Runx2 and osteogenic marker genes, such as alkaline phosphatase (*ALP*), type I collagen (*COL I*), osteocalcin (*OC*) and osteoprotegerin (*OPG*), followed by extracellular matrix mineralization [22], [23]. Several experimental genetic studies on animals and humans have shown Wnt/β-catenin signaling plays an important role in bone mass [24]–[27]. β-catenin, a key regulator of Wnt/β-catenin signaling, is able to induce osteogenic differentiation of osteoblast precursor cells, and reduce apoptosis of mature osteoblast by directly stimulating *Runx2* gene expression [28]. Wnt/β-catenin signaling has also been shown to inhibit adipocyte differentiation of mesenchymal precursor cells [29], [30], which is correlated with the clinical observation of PMOP [31]. Inhibition of glycogen synthase kinase-3β (GSK-3β), which in turn upregulates Wnt/β-catenin signaling, results in increased levels of bone formation in ovariectomized (Ovx) mice and suggests that Wnt/β-catenin signaling may play a role in development of PMOP [32].

This study was performed to investigate the effects of cyclin G2 on osteogenic differentiation and its potential role in estrogen deficiency-induced down-regulation of osteogenic differentiation. Pluripotent mesenchymal precursor cell line C2C12 was utilized, which has been widely used as an *in vitro* model system to assess osteoblast differentiation [33], [34]. Furthermore, we explored the underlying mechanisms of cyclin G2 to suppress estrogen-mediated osteogenesis.

## Materials and Methods

### Cell lines, cell culture and treatment with 17β-estradiol

Mouse pluripotent mesenchymal precursor cell line C2C12 and GP-293 packaging cell line were cultured in Dulbecco's modified Eagle's medium (DMEM, GIBCO, Grand Island, NY) supplemented with 10% fetal bovine serum (FBS) and antibiotics at 37°C in humidified incubators with 5% CO_2_ and 95% air. To test the effects of estrogen, C2C12 cells were seeded overnight and the growth medium was exchanged with steroid-free medium consisting of a phenol red-free DMEM (GIBCO), 10% charcoal-stripped FBS and 1% antibiotics, and cultured for 24 h. The cells were then treated with 100 nM 17β-estradiol (E2, Sigma-Aldrich, USA) at indicated time periods. Dimethyl sulphoxide (DMSO, Sigma-Aldrich) was used to dissolve E2, and DMSO-alone was used as a negative control. The culture medium was changed twice a week.

### Plasmid constructions and transient gene transfection

*CCNG2* cDNA was amplified and cloned into the *Eco*R I and *Bam*H I sites of pLXSN retroviral vector (CLONTECH, CA, USA) and pCMV-3×FLAG 7.1 (Sigma–Aldrich), and pCMV-3×FLAG-BAP was purchased from Genepharma and used as a negative control.

Cyclin G2 expression in cell was induced by performing a transient gene transfection using Lipofectamine 2000 (Invitrogen, CA, USA) according to the manufacturer's recommendations. Briefly, cells were seeded at a density of 2×10^5^ cells per well in a 6-well plate and cultured in DMEM containing 10% FBS to reach 70–80% confluence prior to transfection. Lipofectamine 2000 (10 µl) and DNA (4 µg) were separately diluted in 250 µl serum-free DMEM and combined after 30 min of incubation at room temperature. The DNA-Lipofectamine 2000 complexes were added to cells. After 6 h of transfection, the transfection medium was replaced with normal DMEM containing 10% FBS.

### Recombinant retroviral generation and cell infection

A recombinant retrovirus carrying cyclin G2 was produced for higher and longer expression of ectopic cyclin G2 in target cells. Briefly, GP-293 packaging cell line was plated in 100-mm dishes, followed by co-transfection with the recombinant retroviral vector and pVSV-G (CLONTECH) using Lipofectamine, and cultured for 48 h. Following this, supernatants containing the retroviral particles were collected, filtered through a 0.45-µm filter membrane, and either used immediately or stored at −80°C. To infect cells, the retroviral particles plus polybrene (4 µg/ml, Sigma–Aldrich) were overlaid onto cell cultures for 24 h–48 h and exchanged with complete medium or OS-medium. To maintain ectopic cyclin G2 expression in C2C12 cells when induced for ALP and ARS assay, cells were infected every 3 days.

### In-vitro osteogenic differentiation assays

To induce osteogenic differentiation of C2C12 cells, the cells were seeded into different sized wells and cultured with normal culture media. Upon reaching more than 70% confluence, C2C12 cells were treated with osteogenic supplemental (OS) medium containing 10^−8^ M dexamethasone, 10 mM β-glycerophosphate disodum salt hydrate, and 50 g/ml L-ascorbic acid-2-phosphate (both from Sigma-Aldrich) in complete or steroid-free medium. Cells were collected for semi-quantitative RT-PCR and Western Blot after 48 h of induction. ALP activity were measured on day 7 and mineralization assays on day 14.

### RNA isolation and semi-quantitative RT-PCR

Total RNA was isolated from cultured cells using a TRIzol reagent (Invitrogen) according to the manufacturer's instructions. After quantification of the RNA samples, cDNA was synthesized from 1 µg of total RNA using a TaKaRa RNA PCR kit (AMV) Ver.3.0 (TaKaRa, Dalian, China) and subjected to a semi-quantitative RT-PCR using rTaq (TaKaRa) according to the manufacturer's instructions. PCR primer sequences for amplification of mouse *Ccng2* was performed using the following primer pair: forward, 5′-TGTCTAGCAGAGTATTCTTC-3′ and reverse, 5′- TGTCTGAGCCACTTGGAAG-3′. Other primers were described previously: mouse *Runx2* [35], as well as mouse *Opg*, mouse *Alp*, mouse *Oc and* mouse *Gapdh* [34]. Mouse *Gapdh* was used as an internal control. The above primers were all synthesized by Sangon Biotech (Shanghai, China). The number of cycles for semi-quantitative RT-PCR was chosen to remain the amplification well within the linear range as assessed by densitometry. The PCR products were electrophoresed on a 2% (w/v) agarose gel and stained with ethidium bromide. Gels were scanned and Quantificated by densitometry using the Quantity-One software (Bio-Rad, CA, USA). Data were individually normalized to the mean of the relative expression of mouse *Gapdh*.

### Total and nuclear protein extraction and Western Blot

Cells were lysed for total protein extraction using a radio immunoprecipitation assay (RIPA) buffer (50 mM Tris–HCl buffer, 1% Triton X-100, 150 mM NaCl, 0.1% SDS, 1 mM ethylenediaminetetraacetic acid (EDTA), 1% sodium deoxycholate) in the presence of Protease Inhibitor Cocktail and PhoSTOP Phosphatase Inhibitor Cocktail (both from Roche, Basel, Switzerland) for 30 min, then cleared by centrifugation at 4°C. Nuclear extracts were isolated with a Nuclear and Cytoplasmic Protein Extraction Kit (Bioteke, Beijing, China), according to the manufacturer's instructions. Both total and nuclear extracts were quantified using a BCA assay kit (KeyGEN, Nanjing, China), then resolved by sodium dodecyl sulfate polyacrylamide gel electrophoresis (SDS-PAGE) and transferred onto PVDF membranes. These membranes were the blotted with appropriate primary antibodies and horseradish peroxidase-coupled secondary antibodies, and visualized using chemiluminescence (DNR Bio-Imaging Systems, Jerusalem, Israel) and x-ray films. For Western Blot analysis, β-tubulin and lamin B were used as loading control. Antibodies were obtained from various sources, including Sigma–Aldrich (anti-β-tubulin, anti-β-catenin and anti-CCNG2), Abcam (Cambridge, MA, USA) (anti-CCNG2 and anti-Runx2), Sangon Biotech (anti-ALP and anti-lamin B) and Santa Cruz (anti-CCND1 and anti-c-Myc).

### Detection of ALP activity in cells

After being treated with osteogenic differentiation medium for 7 days, cells were rinsed three times with phosphate buffered saline (PBS) followed by homogenization in an alkaline lysis buffer (20 mM Tris–HCl, pH 7.5, 1% Triton X-100, and 150 mM NaCl) and centrifuged. The supernatant of lysate was then subjected to detection of an ALP activity by measured the rate of conversion of p-nitrophenyl-phosphate (Beyotime, Beijing, China) according to the manufacturer's protocol. Total protein concentration of cell lysates was measured using a BCA kit, and ALP activity was normalized for total protein content in each well.

### Alizarin red S (ARS) staining in detection of mineralization in cells

The formation of calcium deposition in C2C12 cells was determined using ARS staining as described previously [36]. In particular, the cell monolayer were washed with PBS, and then fixed with 100 µl of 100% methanol for 10 min at room temperature. Cells were then stained with 40 mM ARS solution for 10 min and washed three times with PBS. Mineral nodules were documented by photomicrographs from each well.

### Animals and experiments

The protocols for animal use and care were approved by the Animal Care and Use Committee of China Medical University. Twelve six-week-old virgin female C57BL/6 mice were obtained from Laboratory Animal Center of China Medical University and randomly divided into two groups: the eneration of bilateral Ovx mice and sham-operated (Sham) mice that were performed under general anesthesia as described previously [37]. Mice were housed in single cages and kept in a controlled environment for three months to induce osteoporotic conditions. Femora bone samples were collected for further analysis. All surgeries were performed under sodium pentobabital anesthesia, and all efforts were made to minimize suffering.

### Bone mineral density measurement

Mice were anesthetized with isoflurane and placed in a prone position. The whole body bone mineral density (BMD) was determined by dual-energy X-ray absorptiometry (DXA) (Norland XR-36, WI, USA) using software specifically designed for BMD measurement of small animal measurement *in vivo*. The BMD values were expressed as grams per square centimeter.

### Bone protein preparation

Femoral bone was collected and prepared for protein extraction. In general, the femora of mice were dissected free of all connective tissues, and bone marrow were flushed away then crushed using a mortar and pestle in liquid nitrogen. The lysates were collected in 500 µl RIPA buffer for total protein extraction in presence of Protease Inhibitor Cocktail and PhoSTOP Phosphatase Inhibitor Cocktail. The homogenates were centrifuged at 12,000 g at 4°C for 20 min. Resultant supernatant was quantified by a BCA assay and subjected to Western Blot analysis.

### Immunohistochemistry

Femoral bone was fixed with 70% ethanol, decalcified in 10% EDTA-2Na solution for 3 weeks at 4°C, then dehydrated in graded ethanol, and embedded in paraffin. The embedded bone tissues were cut into 5 µm sections, then deparaffinized in toluene and rehydrated with ethanol. SABC staining system (Boster, Wuhan, China) was used to perform immunohistochemical staining. Endogenous peroxidase activity was eliminated by preincubation in methanol with 3% H_2_O_2_ for 10 min. Antigen retrieval was performed by using a pressure cooker and EDTA buffer at 100°C for 2 min. The sections were incubated in 5% BSA in phosphate-buffered saline (PBS) for 20 min to block nonspecific binding sites, followed by incubation with a rabbit polyclonal anti-cyclin G2 antibody (Abcam) diluted 1∶250 overnight at 4°C. After washing in PBS, the sections were then incubated with biotinylated anti-rat secondary antibody (Boster, Wuhan, China) diluted 1∶200 for 20 min and with peroxidase for 10 min. Diaminobenzidine was used as the substrate for color development and visualization under the a Nikon microscope (Nikon E600, Nikon Company, Japan) at indicated magnifications. PBS was used to replace the primary antibody in negative controls.

### Statistical analysis

Data are shown as mean ± SEM of data from at least three separate experiments, each performed with triplicate samples. Statistically analyses were conducted using Student's *t*-test, one- or two-way ANOVA followed by appropriate pairwise post hoc tests with correction for multiple comparisons. Differences were considered statistically significant if *P*<0.05.

## Results

### Cyclin G2 is involved in estrogen-regulated osteogenesis *in vivo* and *in vitro*

**Figure 1:**
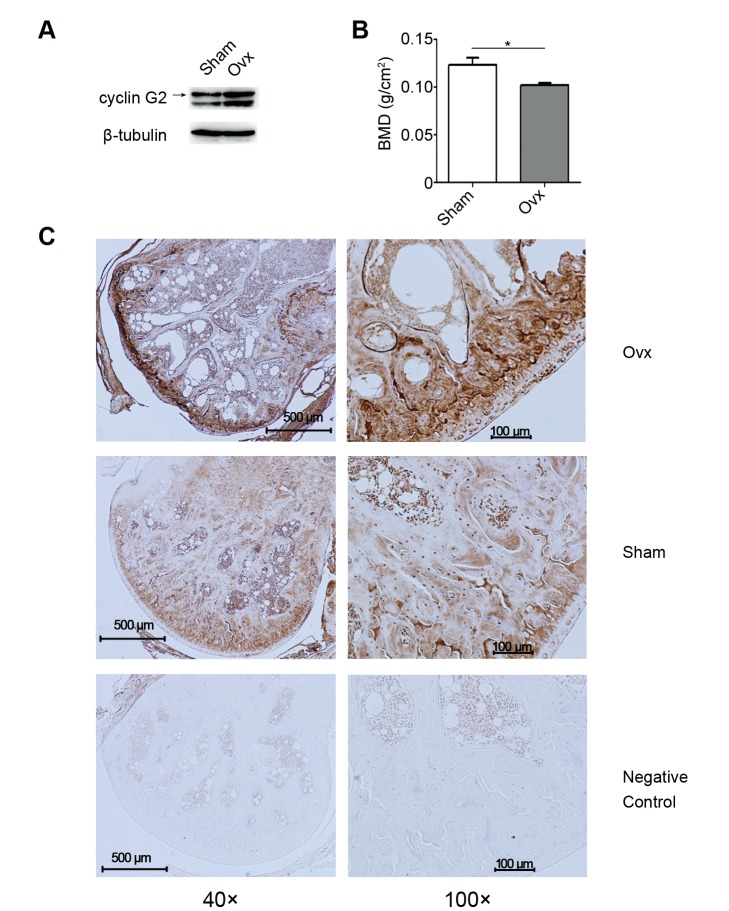
Cyclin G2 is involved in estrogen-deficency induced osteogenesis *in vivo*. (A) Western Blot analysis of cyclin G2 in the femora bone protein isolated from Sham and estrogen-deficient Ovx mice. The results shown are representative of those obtained from three groups of mice. (B) Bone mineral density of Sham and Ovx mice three months after surgery (Six mice per group. The data represent Mean ± SD of six mice per group. *P<0.05 using ANOVA analysis). (C) Immunohistochemical staining of cyclin G2 expression in femora of Sham and Ovx mice. Animal experiments and immunohistochemical staining were conducted as described in *Materials and Methods*. Typical images are shown at two different magnifications: left, 40×; right, 100×. PBS was used to replace the primary antibody as a negative control.

In previous studies [12], cyclin G2 was reported to be down-regulated by estrogen, which deficiency is known to induce bone loss and leads to PMOP. We first determined whether cyclin G2 is involved in this process. Ovx mice and Sham mice were generated and femoral bones were isolated for protein extraction and inmmunohistochemical staining. Expression of cyclin G2 proteins in bone tissues of Ovx mice vs. Sham mice were analyzed by Western blot and inmmunohistochemical staining. It was found that expression of cyclin G2 was up-regulated in the bone of Ovx mice, which is known to be estrogen deficient, accompanied with decreased bone density (Fig. 1A–C). This finding suggests a role of cyclin G2 in estrogen-regulated osteogenesis *in vivo*.

**Figure 2:**
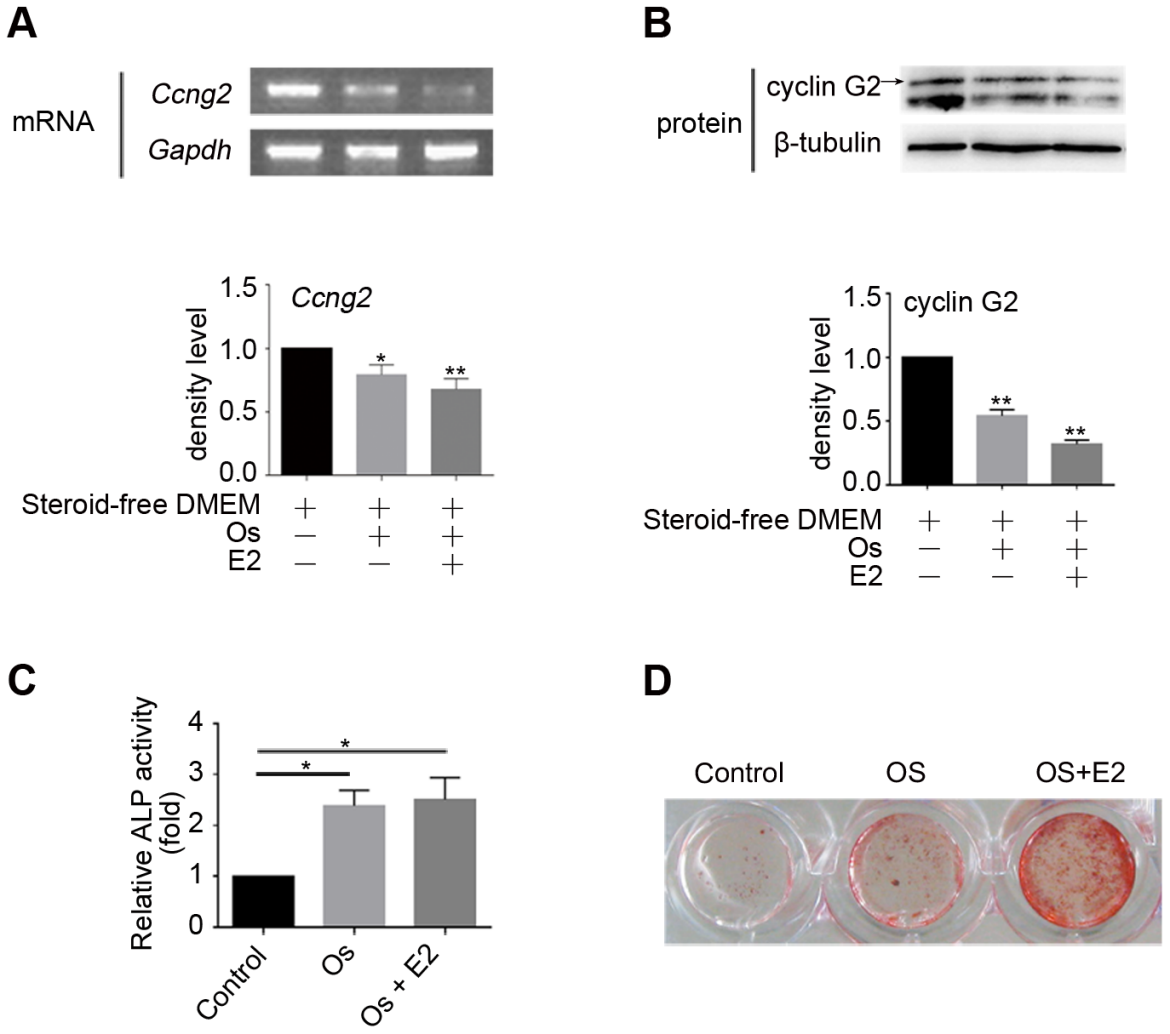
Cyclin G2 is down-regulated in estrogen-mediated osteogenesis *in vitro*. (A and B) Semi-quantitative RT-PCR cyclin G2 mRNA expression and Western Blot analysis of cyclin G2 protein level in steroid-free OS-medium cultured C2C12 cells with or without E2 treatment. C2C12 cells were induced to osteogenic differentiation by steroid-free OS-medium treated with E2 (100 nM) or DMSO. Total RNA and protein were extracted after 48 h of induction, followed by semi-quantitative RT-PCR and Western Blot analysis of cyclin G2. The relative integrated density of each band was digitized by Quantity One. Results are shown as mean ± SEM of data at least three separate experiments, each performed with triplicate samples. *P<0.05 and **P<0.01 vs. control groups by one-way ANOVA. (C and D) ALP activity (Results are shown as mean ± SEM of data at least three separate experiments, each performed with triplicate samples. *P<0.01 vs. control groups by one-way ANOVA) and mineralization (ARS staining) confirmed osteogenic differentiation in C2C12 cells induced by steroid-free OS-medium and E2. Cells were treated as in A, and the amount of ALP activities on day 7 and calcium deposition on day 14 was determined as described under *Materials and Methods*.

We further assessed the expression of cyclin G2 in OS-medium treated C2C12 cells with or without E2. Both mRNA and protein expression of cyclin G2 was reduced by OS-medium treatment, while the inhibition expression stimulated by E2 exhibited milder bands for cyclin G2(Fig. 2A and B). We then confirmed ALP activity and calcium accumulation in the osteogenically differentiated C2C12 cells and observed that OS-medium with or without E2 induced C2C12 cells to osteogenesis (Fig. 2C and D). These manners were inversely correlated with expression of cyclin G2 and suggest that cyclin G2 has a potential role in the regulation of osteogenesis *in vitro*, especially when regulated by estrogen.

### Cyclin G2 suppresses osteogenic differentiation in pluripotent mesenchymal precursor cell line C2C12

**Figure 3:**
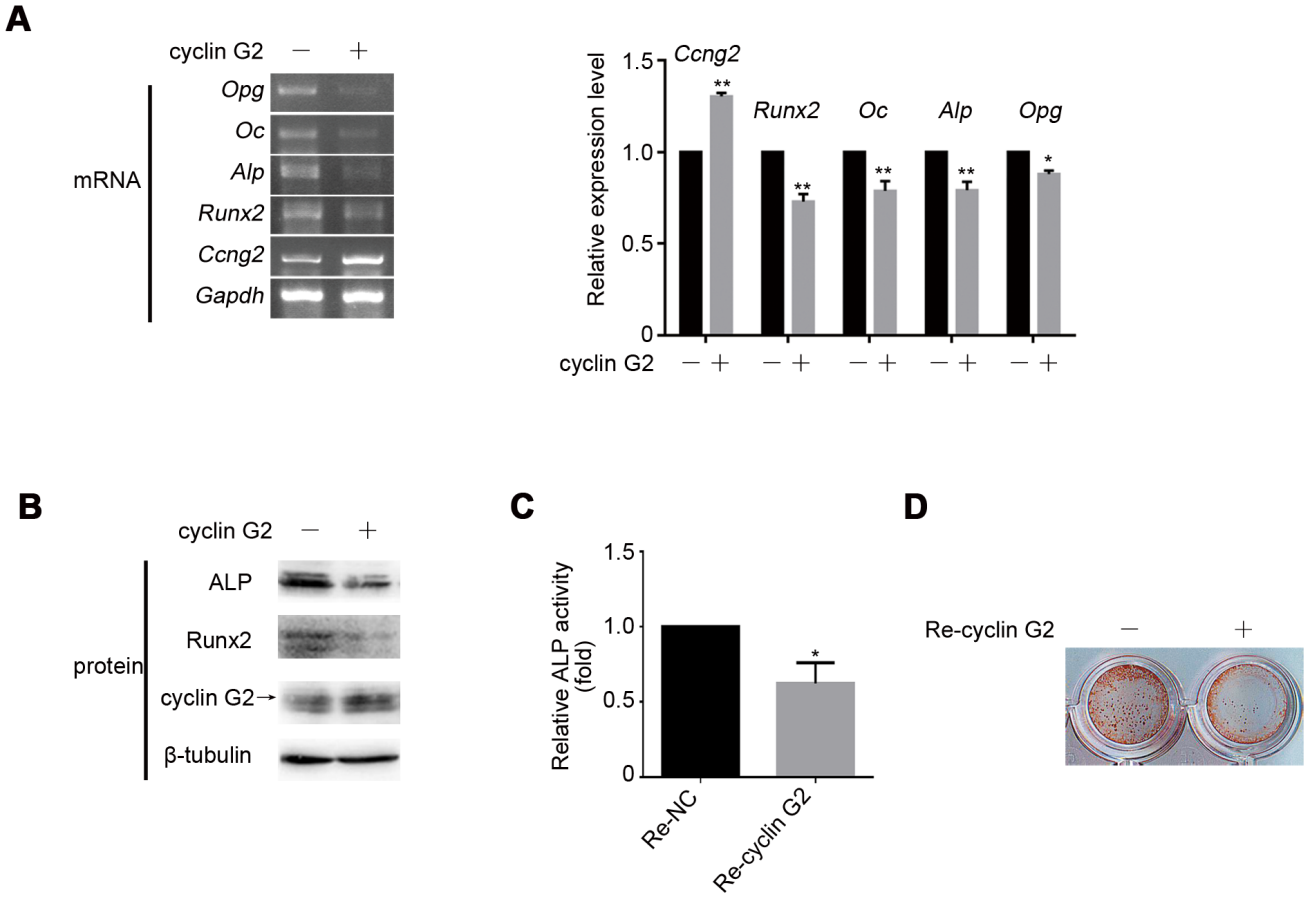
Inhibitory effect of cyclin G2 on osteogenic differentiation of pluripotent mesenchymal precursor C2C12 cells. (A) Semi-quantitative RT-PCR analysis of the inhibition effect of cyclin G2 on osteogenic marker expressions (i.e., *Runx2*, *Oc*, *Alp* and *Opg*). The relative integrated density of each band was digitized by Quantity One. Results are shown as mean ± SEM of data at least three separate experiments, each performed with triplicate samples. *P<0.05 and **P<0.001 vs. control groups by two-way ANOVA. (B) Western Blot analysis of Runx2 and Alp after cyclin G2 overexpression. (C and D) ALP activity (Results are shown as mean ± SEM of data at least three separate experiments, each performed with triplicate samples. *P<0.01 vs. control groups by unpaired *t-test*) and mineralization (ARS staining) were significantly decreased in cyclin G2 overexpression C2C12 cells. C2C12 cells were transfected with cyclin G2 expression vector or infected with recombinant retrovirus encoding cyclin G2 for 24 h, and exchanged for OS-medium to induce osteogenic differentiation. After 48 h of induction, cells were harvest for total mRNA and protein extraction, followed by semi-quantitative RT-PCR and Western Blot analysis. The amount of ALP activities on day 7 and calcium deposition on day 14 was determined as described under *Materials and Methods*.

To directly address the effect of cyclin G2 in osteogenic differentiation, C2C12 cells was infected with a recombinant retrovirus carrying cyclin G2 for higher and longer expression of ectopic cyclin G2 and induced to osteogenesis. The ectopic expression of cyclin G2 was confirmed by semi-quantitative RT-PCR and Western Blot (Fig. 3A and B). Cyclin G2 overexpressed cells showed down-regulation of osteogenic marker genes (e.g., *Runx2, Alp, Oc* and *Opg*) expression (Fig. 3A). Using Western blot we confirmed the inhibitory effects of cyclin G2 on Runx2 and Alp protein levels (Fig. 3B). ALP activity was inhibited in cyclin G2 overexpressing cells compared to cells infected with the control retrovirus (Fig. 3C). ARS staining also showed a decrease in calcium accumulation by ectopic cyclin G2 expression (Fig. 3D). These data confirmed that cyclin G2 overexpression was able to inhibit the osteogenic differentiation of C2C12 cells.

### Cyclin G2 regulates osteogenic differentiation through Wnt/β-catenin signaling pathway

**Figure 4:**
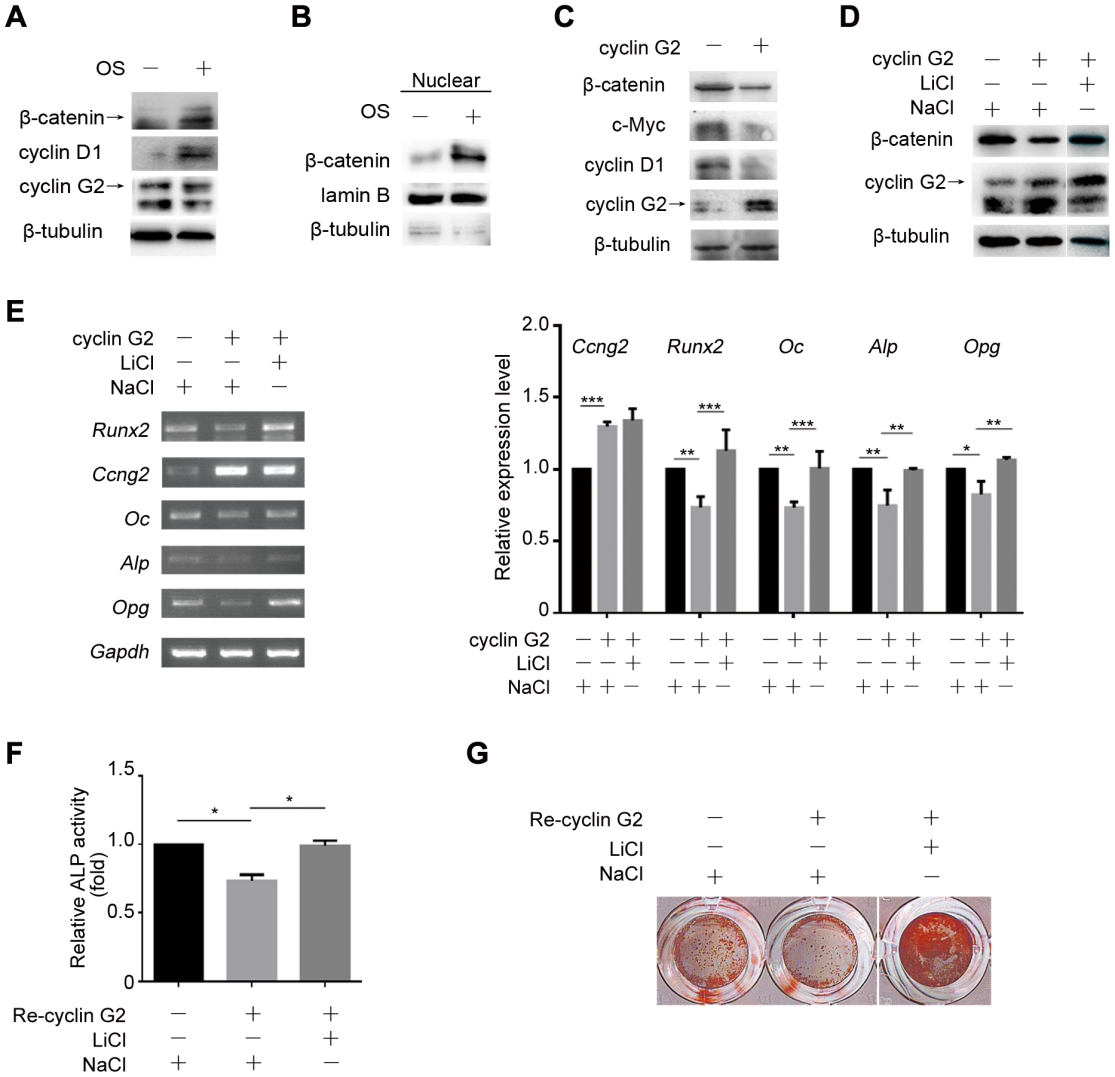
Cyclin G2 inhibits C2C12 cells osteogenic differentiation through antagonizing Wnt/β-catenin Signaling. (A) Western Blot analysis of β-catenin, cyclin D1 and cyclin G2 expression in OS-medium induced C2C12 cells. (B) Western Blot analysis of β-catenin expression in nuclear fraction in C2C12 cells induced by OS-medium. (C) Western Blot analysis of β-catenin, c-Myc and cyclin D1 in cyclin G2-overexpressed C2C12 cells. C2C12 cells were transfected with cyclin G2 expression vector for 48 h, and harvest for total protein extraction followed by Western Blot analysis. (D) Western Blot analysis of β-catenin expression in cyclin G2-overexpressed C2C12 cells that treated with LiCl or NaCl as a negative control. C2C12 cells were transfected with cyclin G2 expression vector, then induced in OS-medium exposed to LiCl (2.5 mM) or NaCl (150 mM). Total protein was harvest after 48 h of induction for Western Blot analysis. (E) Semi-quantitative RT-PCR analysis of osteogenesis differentiation markers (*Runx2*, *Alp*, *Oc* and *Opg*) in cyclin G2-overexpressed C2C12 cells that treated with LiCl or NaCl as a negative control. Total RNAs were extracted from C2C12 cells after transfected and treated as in D to perform Semi-quantitative RT-PCR analysis. The relative integrated density of each band was digitized by Quantity One. Results are shown as mean ± SEM of data at least three separate experiments, each performed with triplicate samples. *P<0.05, **P<0.01 and ***P<0.001 vs. control groups by two-way ANOVA. (F and G) ALP activity (Results are shown as mean ± SEM of data at least three separate experiments, each performed with triplicate samples. *P<0.05 by one-way ANOVA) and mineralization (ARS staining) of cyclin G2-overexpressed C2C12 cells that treated with LiCl or NaCl. C2C12 cells were infected with recombinant retrovirus carrying cyclin G2 cDNA for 42 h and then treated as in D, followed the measurement of ALP activity on day 7 and calcium deposition on day 14.

Wnt/β-catenin signaling pathway has been shown to play a major role in osteogenic differentiation. We first determined whether Wnt/β-catenin signaling is involved in cyclin G2-suppressed osteogenesis. Results showed that expression of β-catenin, the key mediator of the Wnt/β-catenin signaling pathway, and its target cyclin D1 was induced in C2C12 cells by OS-medium accompanied by reduced expression of cyclin G2 (Fig. 4A). The increased nucleus fraction of β-catenin protein level further confirmed the activation of Wnt/β-catenin (Fig. 4B). Furthermore, overexpression of cyclin G2 by transient gene transfection inhibited expression of β-catenin protein and its target gene cyclin D1 and c-Myc protein expression compared to the control cells (Fig. 4C). Thus, we hypothesized that the mechanism by which cyclin G2 inhibits C2C12 cells osteogenic differentiation may be through suppression of Wnt/β-catenin signaling.

To determine whether activation of Wnt/β-catenin signaling is sufficient to interfere with cyclin G2-inhibited osteogenesis, we examined the effect of LiCl, an activator of Wnt/β-catenin signaling activity, on cyclin G2-regulated osteogenic differentiation of C2C12 cells. It was observed that LiCl treatment was able to reverse the down-regulated β-catenin expression by ectopic expression of cyclin G2 as well as the down-regulated osteogenic marker genes expression (Fig. 4D and E), ALP activity and mineralization (Fig. 4F and G). These findings collectively demonstrate that cyclin G2 regulates C2C12 cells osteogenic differentiation through Wnt/β-catenin signaling.

### Cyclin G2 inhibits estrogen-regulated osteogenesis

**Figure 5:**
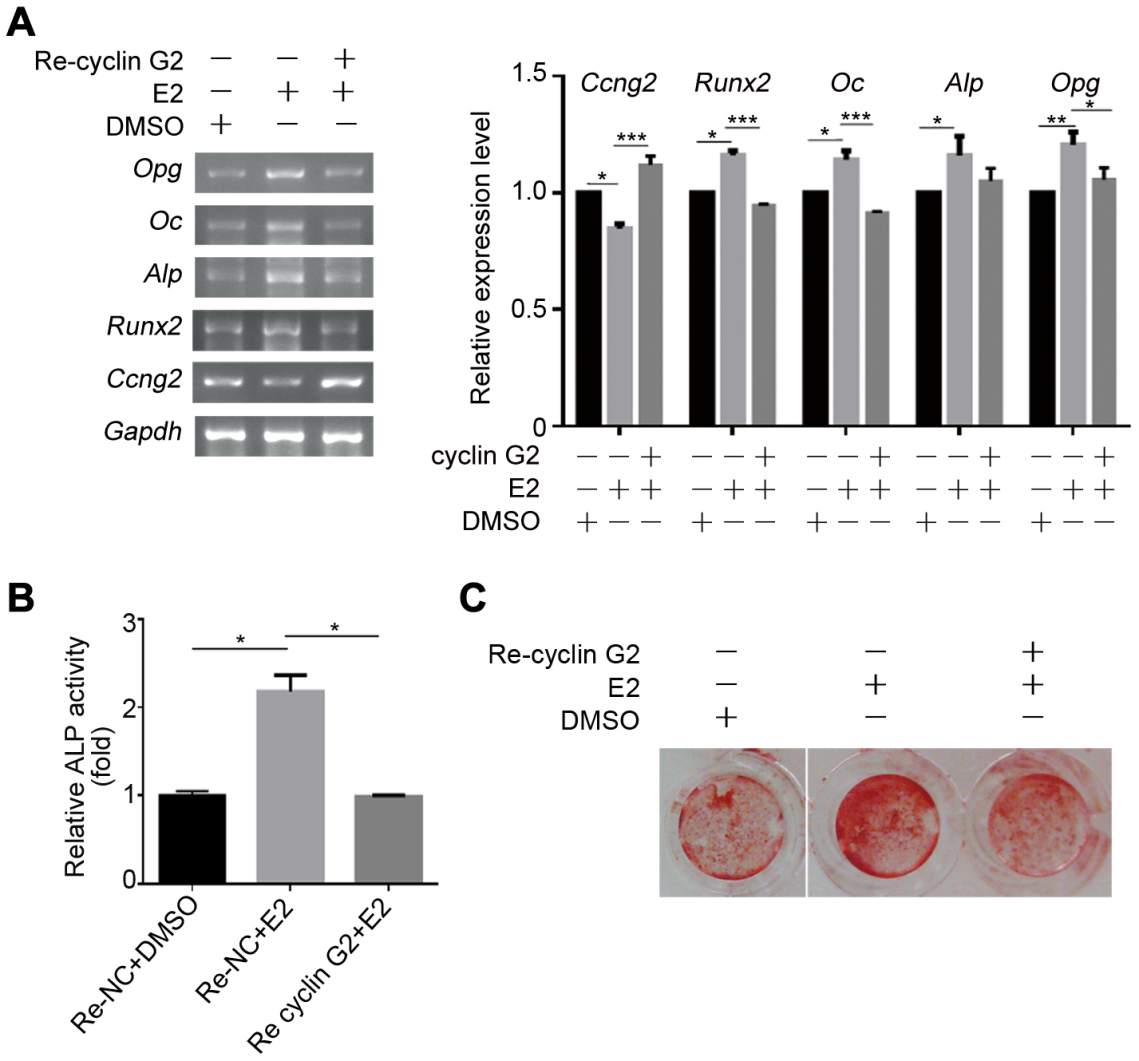
Cyclin G2 inhibits effects of estrogen on osteogenic differentiation. (A) Semi-quantitative RT-PCR analysis of osteogenic differentiation marker genes in C2C12 cells that were transfected with cyclin G2 expression or control vector for 6 h, and exchanged steroid-free OS-medium with E2 (100 nM) or DMSO. Total RNA was extracted after 48 h of induction, followed by semi-quantitative RT-PCR analysis. The relative integrated density of each band was digitized by Quantity One. Results are shown as mean ± SEM of data at least three separate experiments, each performed with triplicate samples. *P<0.05, **P<0.01 and ***P<0.001 between the indicated groups by two-way ANOVA. (B and C) ALP activity (Results are shown as mean ± SEM of data at least three separate experiments, each performed with triplicate samples. (*P<0.001 between the indicated groups by one-way ANOVA.) and mineralization (ARS staining) in C2C12 cells infected with recombinant retrovirus encoding cyclin G2 or control gene for 24 h, and then exchanged for steroid-free OS-medium with E2 (100 nM) or DMSO to induce osteogenic differentiation. ALP activities were measured on day 7 and calcium deposition was on day 14.

In order to elucidate the specific contribution of cyclin G2 in estrogen-regulated osteogenesis, C2C12 cells was overexpressed cyclin G2 by transient transfection or recombinant retroviral carrying *CCNG2* and then exposure to E2. Expression of the osteogenic maker genes, ALP activity and mineralization, were induced by E2, while cyclin G2 overexpression impaired the effects of E2 on these cells (Fig. 5A–C). Altogether, the present study suggests that cyclin G2 functions as an endogenous suppressor of osteogenic differentiation and may play an essential role in estrogen regulated osteogenesis through inhibition of Wnt/β-catenin signaling.

## Discussion

In this study, we observed that cyclin G2 was involved in estrogen regulated osteogenesis *in vivo* and *in vitro*. Further investigation confirmed that cyclin G2 suppresses osteogenic differentiation through inhibition of Wnt/β-catenin signaling pathway. Finally, it was demonstrated that cyclin G2 inhibits estrogen-regulated osteogenesis. Therefore, cyclin G2 suppresses estrogen-regulated osteogenesis uses multiple steps of the molecular process including the inhibition of Wnt/β-catenin signaling pathway. To the best of our knowledge, the current study presents the first evidence that cyclin G2 serves as a negative regulator of both osteogenesis and Wnt/β-catenin signaling. Further studies will focus on investigating functions of cyclin G2 in estrogen-mediated osteogenesis *in vivo* and the clinical significance of this gene pathway on participation the development of PMOP.

Indeed, dysregulation of osteogenesis has been linked to various diseases of bone including osteoporosis [38], a common phenomenon in postmenopausal women with reduced bone mass due to estrogen deficiency [39]. Estrogen and its gene pathway play an important role in osteogenic differentiation and contribute to the progression of PMOP. Although signaling cascades that control osteogenesis have recently began to emerge, it is largely unclear how estrogen regulates osteogenic differentiation. Using an animal model of osteoporosis in Ovx mice, which has been widely accepted in the research of PMOP, we showed that cyclin G2 is involved in estrogen-mediated osteogenesis *in vivo*. Specifically, increased expression of cyclin G2 protein was shown in the Ovx mice femora bones as compared to that of the Sham mice. These data were consistent with the *in vitro* data that cyclin G2 is involved in estrogen and OS-medium induced osteogenic differentiation. Our data confirmed results of the previous studies that estrogen inhibited cyclin G2 expression [12]. Moreover, it was found that steroid-free OS-medium without E2 treatment also induced a significant down-regulation of cyclin G2 mRNA and protein. In addition, the inhibitory effect of cyclin G2 on osteogenic differentiation was further confirmed by down-regulation of osteogenic marker genes expression levels, ALP activity and calcium accumulation. These findings suggest that cyclin G2 plays a negative role in osteogenic differentiation and mineralization. To the best of our knowledge, the current study presents the first evidence showing cyclin G2 inhibited osteogenesis.

The underlying molecular signaling responsible for cyclin G2-suppressed osteogenesis was explored. One candidate is Wnt/β-catenin signaling pathway, which has key functions in embryo development, tissue self-renewal, and tumorigenesis [40], [41]. Previous studies showed that lost of cyclin G2 expression was associated with the development of gastric, ovarian, and breast cancers [42]–[44], but activation of Wnt/β-catenin signaling [38], [45], [46] indicated a potential link between them. Indeed, it was demonstrated that both the total and nucleus fraction levels of β-catenin were up-regulated during OS-medium induced osteogenesis of C2C12 cells, but were inversely associated with cyclin G2 expression. Moreover, activation of Wnt/β-catenin signaling by LiCl treatment rescued mRNA levels of the osteogenic differentiation marker genes, ALP activity and mineralization ability, which were suppressed by ectopic cyclin G2 expression. These data combined extend the role of cyclin G2 as an osteogenesis suppressor through suppression of Wnt/β-catenin signaling and its downstream targets.

Furthermore, our data also revealed that overexpression of cyclin G2, which was unable to be inhibited by E2 treatment, at least partly reversed the up-regulation of E2 on osteogenic differentiation *in vitro*. These findings further suggest cyclin G2 as a potential target in the prevention and control of PMOP *in vivo*. To address this possibility, further studies using osteoblast-specific knockout of *Ccng2* will be required to clarify this discrepancy. Even though, this study proposed a working model, in which cyclin G2 inhibits osteogenic differentiation and mineralization through suppressing Wnt/β-catenin signaling and mediated estrogen-regulated osteogenesis *in vitro* and *in vivo*. Future work will aim to further confirm these data by using transgenic mice to provide more evidence of these genes in participation the formation and development of PMOP.
